# Individualized Determination of the Mechanical Fracture Environment After Tibial Exchange Nailing—A Simulation-Based Feasibility Study

**DOI:** 10.3389/fsurg.2021.749209

**Published:** 2021-09-29

**Authors:** Benedikt J. Braun, Marcel Orth, Stefan Diebels, Kerstin Wickert, Annchristin Andres, Joshua Gawlitza, Arno Bücker, Tim Pohlemann, Michael Roland

**Affiliations:** ^1^University Hospital Tuebingen on Behalf of the Eberhard-Karls-University Tuebingen, Faculty of Medicine, BG Hospital Tuebingen, Tuebingen, Germany; ^2^Department of Trauma, Hand and Reconstructive Surgery, Saarland University Hospital, Homburg, Germany; ^3^Department of Applied Mechanics, Saarland University, Saarbruecken, Germany; ^4^Department of Diagnostic and Interventional Radiology, Technical University of Munich, Munich, Germany; ^5^Clinic of Diagnostic and Interventional Radiology, Saarland University Hospital, Homburg, Germany

**Keywords:** non-union, individualized simulation, fracture healing, tibia, simulation

## Abstract

Non-union rate after tibial fractures remains high. Apart from largely uncontrollable biologic, injury, and patient-specific factors, the mechanical fracture environment is a key determinant of healing. Our aim was to establish a patient-specific simulation workflow to determine the mechanical fracture environment and allow for an estimation of its healing potential. In a referred patient with failed nail-osteosynthesis after tibial-shaft fracture exchange nailing was performed. Post-operative CT-scans were used to construct a three-dimensional model of the treatment situation in an image processing and computer-aided design system. Resulting forces, computed in a simulation-driven workflow based on patient monitoring and motion capturing were used to simulate the mechanical fracture environment before and after exchange nailing. Implant stresses for the initial and revision situation, as well as interfragmentary movement, resulting hydrostatic, and octahedral shear strain were calculated and compared to the clinical course. The simulation model was able to adequately predict hardware stresses in the initial situation where mechanical implant failure occurred. Furthermore, hydrostatic and octahedral shear strain of the revision situation were calculated to be within published healing boundaries—accordingly the fracture healed uneventfully. Our workflow is able to determine the mechanical environment of a fracture fixation, calculate implant stresses, interfragmentary movement, and the resulting strain. Critical mechanical boundary conditions for fracture healing can be determined in relation to individual loading parameters. Based on this individualized treatment recommendations during the early post-operative phase in lower leg fractures are possible in order to prevent implant failure and non-union development.

## Introduction

Despite current clinical advances diaphyseal tibial fractures are associated with delayed- and non-union rates of over 10% (1–3). The development of a healing delay is dependent on many factors that often cannot be adequately influenced once the fracture has occurred (4). Of high significance in aseptic cases are vascularity and mechanical fracture environment (5). To determine the relevant mechanical influences on fracture healing numerical modeling and computer simulation has gained increasing interest (6). Based on the initial ideas of Pauwels, Wolff, Perren, and Frost ever more precise mechanical fracture environment boundary conditions for influencing tissue differentiation can now be given (7–12). Despite the differences between the models owed in part to the specifics of the simulations and input characteristics, these approaches and their experimental validation in animal research underscore the great importance of the mechanical environment for fracture healing. Two of the most relevant parameters to determine tissue differentiation during the course of fracture healing are interfragmentary shear strain and tensile or compressive volumetric strain (13, 14). While the clinical use of these simulated parameters has been shown in the case of a patient treated with an external fixator (15), these boundary conditions for hydrostatic pressure and volumetric fracture strain have yet to be applied to a clinical case with internal osteosynthesis. Despite its theoretical relevance especially in cases where failed fracture healing and failure of implant material point toward a high mechanical influence this has not been performed. Current clinical management is largely based on general treatment principles and surgeon experience depending on the applied hardware.

The aim of this study was, thus, to establish a simulation workflow based on clinical imaging data to (1) determine the pre- and post-treatment mechanical fracture environment in a tibial fracture revision case, (2) simulate the associated volumetric strain and octahedral shear strain resulting from patient weight-bearing, and (3) provide a clinical proof-of-concept over the treatment course.

## Materials and Methods

### Patient Case Data

**Figure 1:**
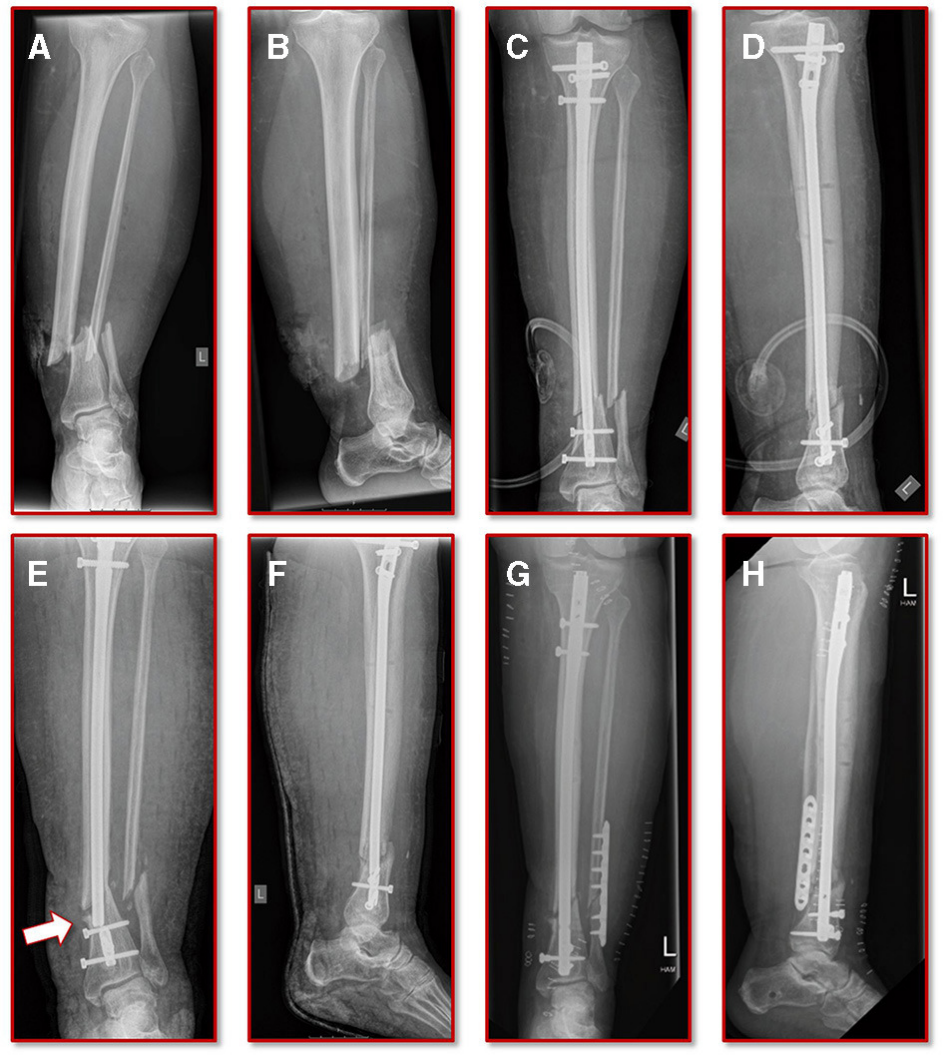
Initial clinical imaging. **(A,B)** a.p. and lateral view of the initial 2° open distal tibia fracture. **(C,D)** a.p. and lateral view after intramedullary tibial nail implantation and split thickness skin grafting, following temporary external fixation debridement and negative pressure wound therapy. **(E,F)** a.p. and lateral view after refracture and implant failure. **(G,H)** a.p. and lateral view after two-step exchange nailing and fibular plate osteosynthesis.

A 55-year old, female patient (height 152 cm, weight 73 kg) was treated for an open distal tibial shaft fracture (Figures 1A,B) with debridement, temporary external fixation, negative pressure wound therapy, unreamed intramedullary tibial nailing (8 mm diameter) (Figures 1C,D) and MESH graft skin closure at an external institution within the span of 4 weeks. Immediate post-operative full weight-bearing was ordered. Approximately 7 weeks after the tibial nail procedure was performed and without further trauma the patient suffered from an implant failure and refracture of the initial situation (Figures 1E,F). She was then referred for treatment to our institution, where after an initial hardware removal, temporary external fixation and histological and microbiological exclusion of infection, a reamed nailing procedure (9 mm nail diameter) and fibular plate osteosynthesis was performed (Figures 1G,H). Again, immediate post-operative full weight-bearing was performed and reached within the inpatient stay, as controlled with plantar pressure measurements (16). To estimate the non-union risk of the patient the Non-Union Risk Development (NURD) Score was calculated as 7 (17). Furthermore, the patient suffered from a severe Neurofibromatosis, that if counted as a chronic condition would increase the NURD score further to 9. The patient consented to the study. Only routine imaging data was used and the study was approved by the local ethics committee.

### Simulation Work Flow

The simulation workflow is divided into three main steps: (1) the geometric model generation based on clinical imaging, (2) the computation of individual biomechanical parameters from patient monitoring, and (3) the performance of the final finite element (FE) simulations and follow-up mechanical data analysis.

**Figure 2:**
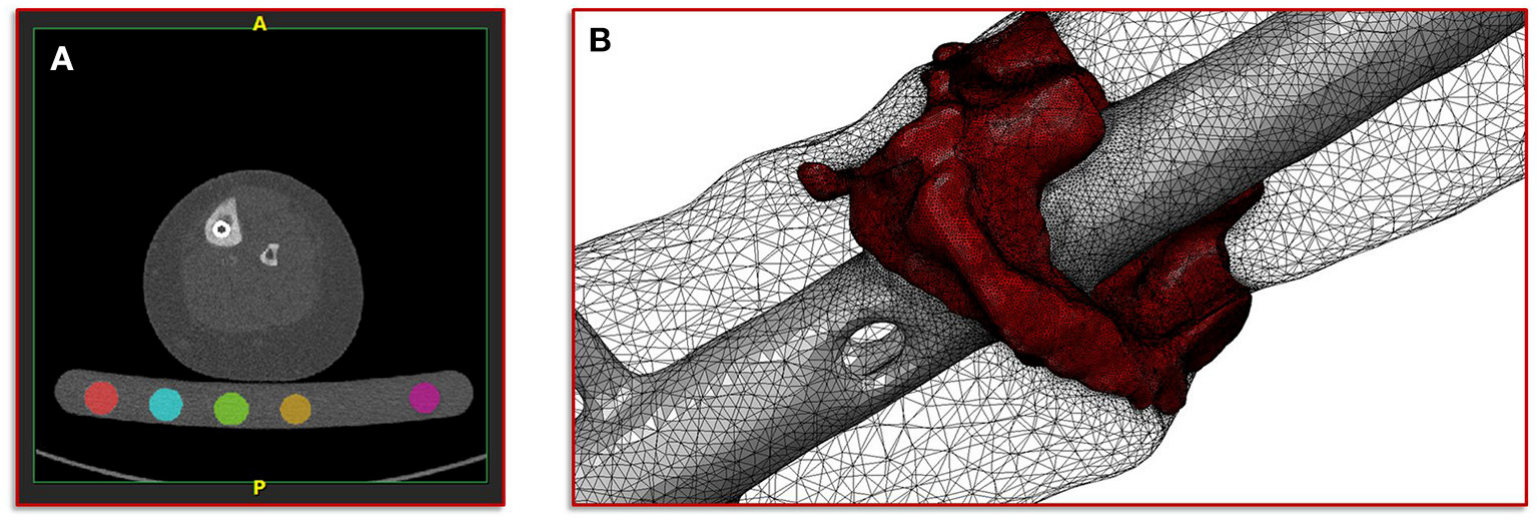
Illustration of the segmentation, image processing and meshing procedure. **(A)** Image shows a typical slice of the CT image stack with segmented rods of the calibration phantom. **(B)** Image shows the reconstructed mesh of the fracture and the intramedullary nail; additionally, the surface mesh of the bone mask is shown transparently.

The basis of the geometric model creation is established on an image processing chain of various algorithmic steps in a semi-automated sequence. The starting point is the dicom image stack of the patient's post-operative CT scan combined with a six-rod bone density calibration phantom (QRM-BDC/6, QRM GmbH Moehrendorf, Germany). The images were segmented into different masks (intramedullary tibial nailing, fracture gap, and bone) with an adaptive threshold procedure with respect to the calibration phantom, supplemented by a morphological close filter with isotropic values and a mask smoothing with a recursive Gaussian filter with anisotropic values. Afterwards, for each segmented mask an island removal, a cavity fill and a fill gaps with a priority order procedure was performed resulting in a high segmentation quality without detectable problems. In addition, the orthopedic trauma surgeons manually controlled the segmentation results of the fracture gap and if necessary corrected them. All image processing steps as well as the generation of the FE meshes were performed in the image processing and model generation software ScanIP (Synopsys, Mountain View, CA, United States). Figure 2 shows a typical slice of the CT image stack with the segmented rods of the calibration phantom.

After segmentation was completed, a high-resolution adaptive FE mesh was created from the individual masks. Since all simulations were performed with the FE analysis and computer-aided engineering software suite Abaqus (Dassault Systemes, Velizy-Villacoublay, France), quadratic tetrahedral FE of type C3D10 with straight lines were chosen. The material parameter assignment for the masks of the intramedullary nailing, the corresponding screws and the fracture gap were chosen as homogeneous materials with standard properties from literature. The Young's modulus for the nail and the screws was set to 108,000 MPa and the Poisson ratio was set to 0.37 (18). For the fracture gap, the values 3 MPa for the Young's modulus and 0.4 for the Poisson ratio (initial connective tissue) were chosen (13).

For the bone mask, material properties with respect to the calibration phantom were defined, in order to derive an empirical elasticity-bone density relationship. For this purpose, a histogram analysis of the individual segmented rods was performed in a first step. Then, by means of a linear regression, a mapping of the Hounsfield units (HU) to the hydroxy apatite values from the calibration phantom was defined. This mapping provides the basis for the gray-scale-dependent definition of the material parameters representing local bone properties (19, 20). Here, an isotropic heterogeneous material was assumed, having a different value for the Young's modulus and a fixed value for the Poisson ratio. This type of material model has shown a good agreement with experimental observations (21, 22). Depending on the local ash density and the equivalent mineral density the mapping for the cortical and the trabecular bone is defined as follows (23, 24):


(1) $$\begin{array}{l} \rho_{ash}=1.22\rho_{eqm}+0.0523\;g/cm^{3} \end{array}$$

(2) $$\begin{array}{l} E_{cort}=10,200\times \rho_{ash}^{2.01}\;MPa \end{array}$$

(3) $$\begin{array}{l} E_{trab}=5,307\times \rho_{ash}+469\;MPa\; \end{array}$$

with a fixed Poisson ratio of ν = 0.30. The ash density relation corresponding to the equivalent mineral density is in accordance with Les et al. (25). All material properties were passed to the FE mesh and stored in the elements of the corresponding masks. Figure 2 shows the mesh of the fracture gap and the intramedullary nailing combined with a transparently visualization of the bone mask.

**Figure 3:**
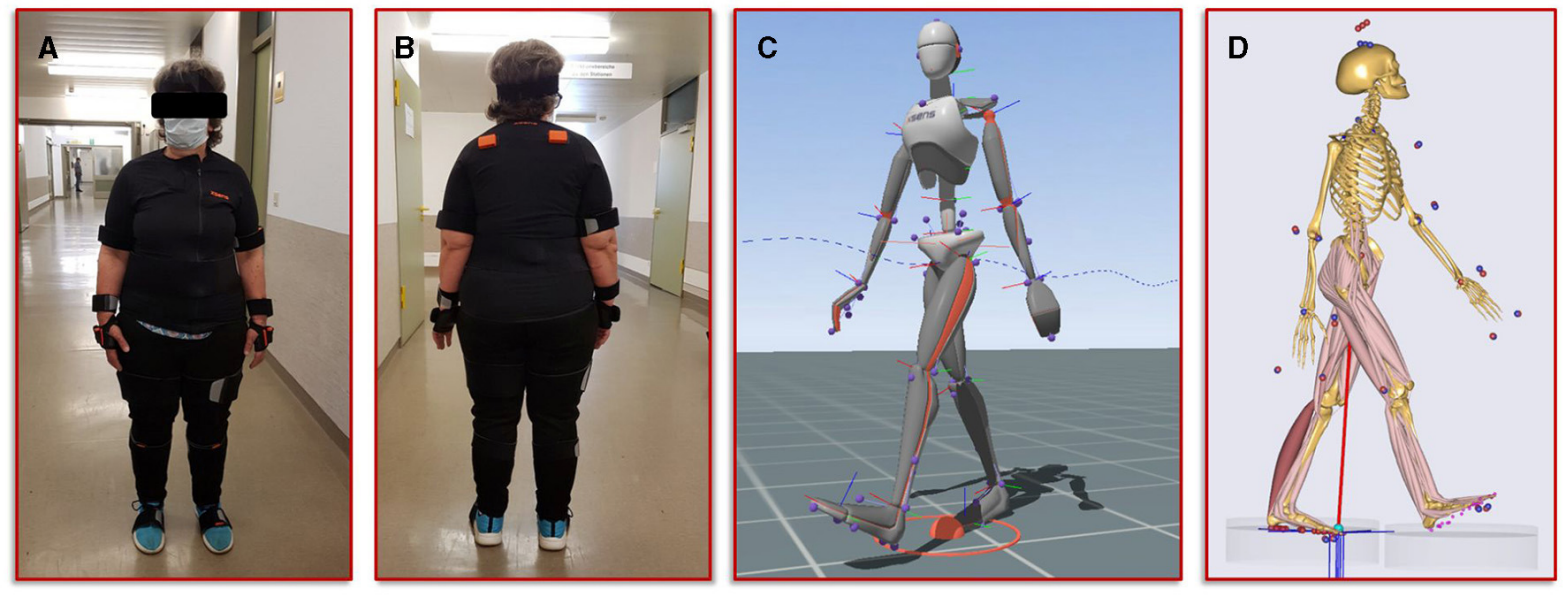
Illustration of the motion capturing workflow for the generation of patient-specific biomechanical simulation parameters. **(A,B)** Setup of the Xsens system and positioning of the sensors on the patient. **(C)** Visualization of the avatar in the Xsens analysis software. **(D)** representation of the musculoskeletal model from AnyBody, scaled with the patient's body measurements and equipped with her motion data.

The second step in our simulation workflow consists of monitoring the patient during normal forward gait by means of the full body motion capturing system Xsens MVN Awinda (Xsens Technology B.V., Enschede, Netherlands). The MVN Awinda system uses 17 wireless sensors, which are fitted on the body with adjustable straps and a specific T-shirt. The T-shirt is used to attach the sensors on the shoulder (two sensors, one on the left and one on the right), one sensor on the sternum and one sensor on the pelvis. Tapes are used to attach the sensors to the biomechanically relevant segments: on the upper arms, on the forearms, on the thighs, on the lower legs and on the feet. Finally, two sensors are attached to both hands with special gloves and one sensor is fixed to the head with a headband. Figure 3 shows photos of the patient wearing the Xsens MVN Awinda system. The system internally measures and processes motion data at 1,000 Hz and provides data at an output rate of 60 Hz in the corresponding evaluation and analysis software Xsens MVN Analyze. The MVN Analyze software allows a comprehensive analysis of the recorded motion data. In addition, the data of the individual biomechanical segments and the joints defined from them were examined with regard to the angles, the movement, the speed, and the acceleration. Subsequently, the motion data were processed in the MVN software into the BVH (Biovision Hierarchy) data format for export to the musculoskeletal simulation environment AnyBody (AnyBody Technology A/S, Aalborg, Denmark). Figure 3 shows the biomechanical avatar of the patient during gait in the MVN Analyze software and the corresponding avatar in the AnyBody software after importing the motion capturing data. The AnyBody modeling system allows the simulation of individual muscle forces, ligament forces, and internal joint contact forces, which are essential for the understanding of the mechanical mechanisms of human movement. To achieve the goal addressed in the present study, a simulation of the tibia that is as individualized and patient-specific as possible, the motion capturing data from the patient's gait was also used to scale the model in AnyBody. These results serve as personalized boundary conditions in the FE simulations. The results from AnyBody, together with the image data-based geometric model and the calibrated material parameters were used in Abaqus for running the FE simulations.

### Generation of the Computational Model of the Failed Treatment

**Figure 4:**
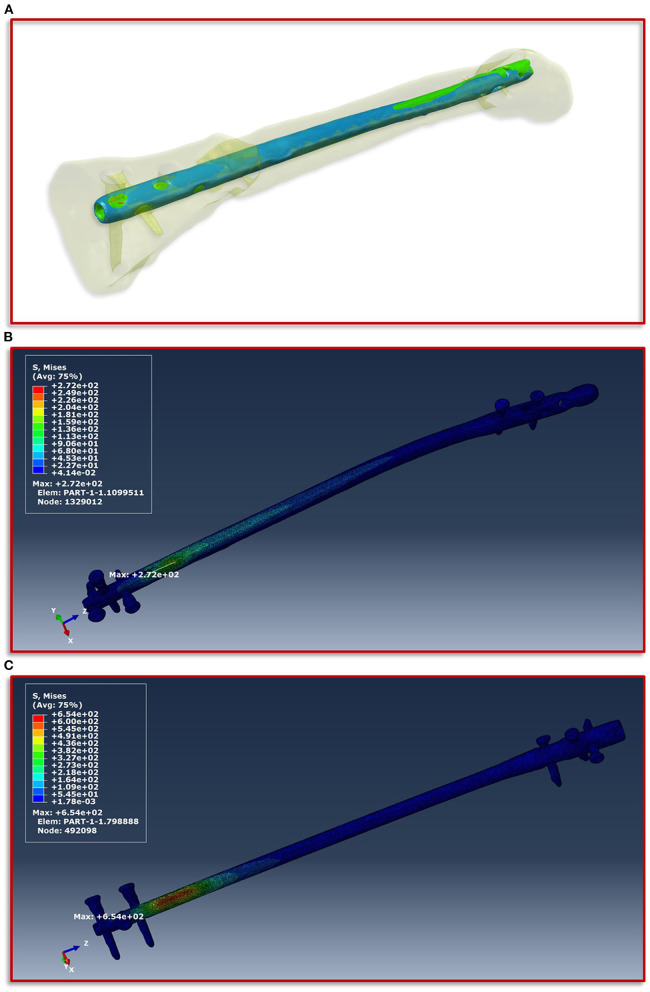
Implant positioning and resulting von Mises stress distribution. **(A)** Positioning of the smaller intramedullary nail of the first treatment (blue) with respect to the current treatment (red). The positioning is aligned with the distal end of the current treatment and follows this in proximal direction. **(B)** Shows the FE simulations of the von Mises stress distribution of the intramedullary nail and the screws of the current treatment and **(C)** of the first failed treatment with the smaller intramedullary nail.

To obtain a simulation model of the failed treatment case of which only conventional radiographs but no CT data existed, a CT scan of an identical intramedullary nail was achieved. This CT scan was segmented in ScanIP and transferred to a computer aided design (CAD) file in stereolithography (stl) format using a mask to surface operation. The surface model was imported into the CT image stack after the exchange of the intramedullary nails. Along with a visual inspection by the treating trauma surgeons the surface model of the first treatment was aligned with the distal end of the new treatment and followed proximally. The positioning at the knee was performed according to the radiographs and the new treatment. Subsequently, the additional screw used in the first failed treatment compared to the new one was also integrated into the model based on the radiographs and the positional locations present in the associated intramedullary nail. In addition, all other screws were also adjusted corresponding to their type and positioning in the model. Then, the same workflow was applied to create the geometric model and associated material parameters, which is described in detail above. Figure 4A illustrates the described positioning of the first failed treatment with respect to the current treatment.

## Results

### Clinical Results

**Figure 5:**
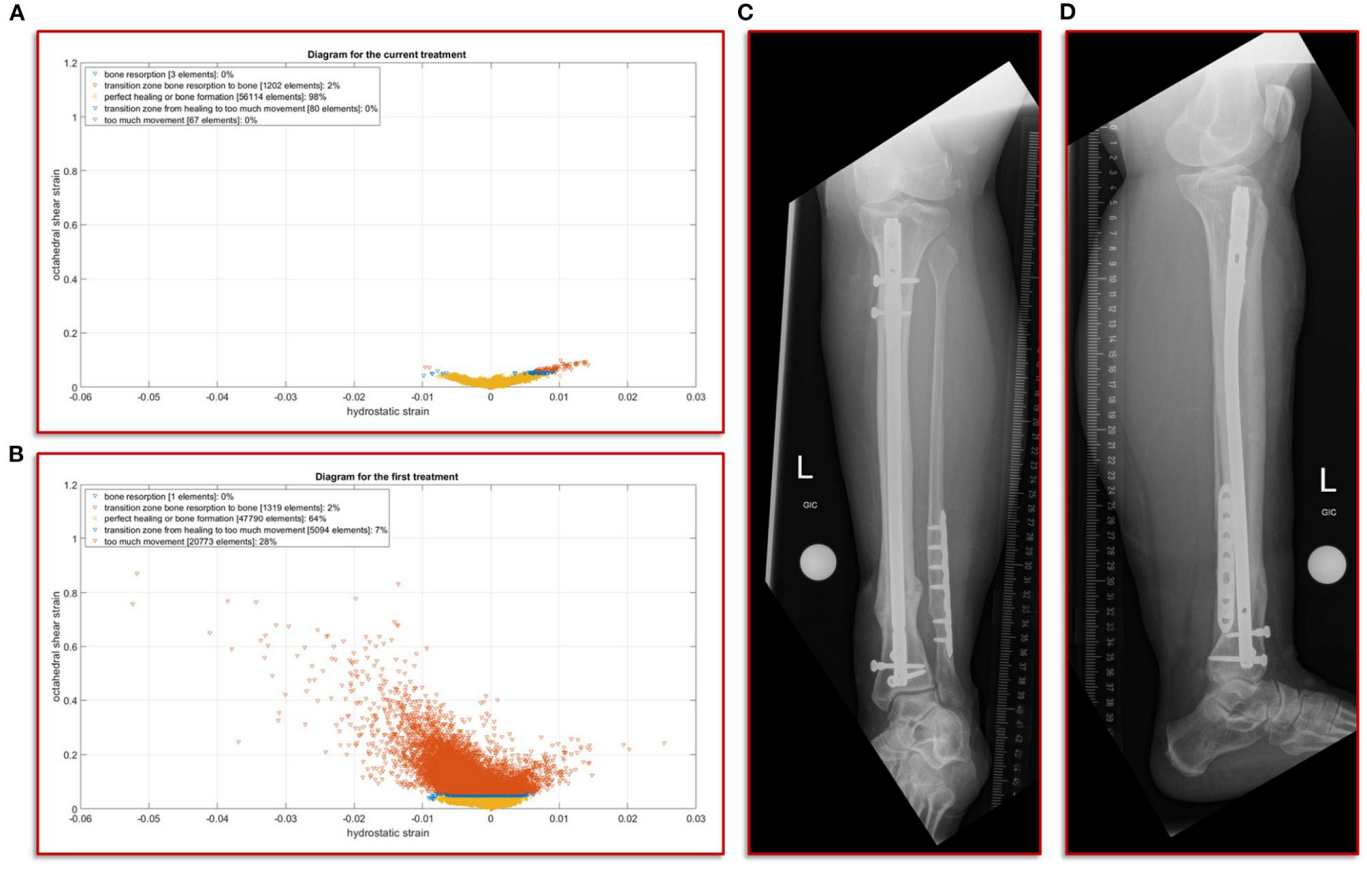
Results of the simulation of the interfragmentary movement with respect to the hypothesis-based correlations between mechanical conditions and types of tissues in a fracture callus (18,29,30,32) **(A)** Shows the results after the revision treatment, **(B)** for the failed treatment with almost 30% of strain above the healing boundary conditions. **(C,D)** a.p. and lateral view at the final follow-up. The fracture is fully healed and the mRUST score is calculated at 15.

The further clinical course of our patient after the exchange nailing procedure was uneventful with undisturbed soft tissue healing and mobilization with full weight-bearing. At the final follow-up at 18 months the patient was walking free, without aids and back to her self-reported activity and pain level as before the initial fracture event. The radiographic controls showed a healed fracture situation with an mRUST score of 15 (Figure 5).

### Simulation of the Von Mises Stress of the Different Intramedullary Nailings

The boundary conditions derived from the musculoskeletal simulations based on the patient's motion capturing data were applied on the knee in the computational model, while on the side of the foot fixed Dirichlet boundary conditions were defined. Figure 4 shows the von Mises stress distribution at the moment of maximum force application during a patient forward step. Here, Figure 4B presents the results for the current treatment, while Figure 4C shows the von Mises stress distribution for the first failed treatment. In both cases, the region with the highest von Mises stress values is almost congruent in the area of the fracture above the most proximal of the distal screws. However, in the case of the first treatment a significantly higher value is shown with a maximum of 654 MPa, as in the case of the current treatment with 272 MPa. If this difference is considered with regard to the tensile strength and the yield strength of titanium alloys in surgical implants, which are specified in the ASTM F1472-14 standard as a minimum of 930 and 860 MPa, this could be a hint as to why the first treatment failed. Especially considering that the relative high value of around 654 MPa occurred with every step the patient took and spatially located closer to the point above the third screw where the mechanical failure occurred.

### Simulation of the Interfragmentary Movement With Respect to the Different Treatments

In this study, we focus on the mechanobiological approach based on the mechanical stimulus, as addressed in the works of Claes and Heigele (13) and Shefelbine et al. (26). In other words, we examine the interplay of octahedral shear strain derived from the deviatoric part of the strain tensor associated with the shape distortion and the volumetric strain connected with the volume change and the hydrostatic pressure (6). This approach is strongly linked to the concept that the level of mechanical deviatoric strains is the main factor determining differentiation of cells and consequently the process of tissue formation and agrees well with the experimental work of Bishop et al. (27), Garcia et al. (28), and Doblaré et al. (29). In order to perform the most accurate analysis of the mechanics within the fracture gap, the strain tensor was read for each individual tetrahedral element of the fracture gap, and the octahedral shear strain as well as the volumetric strain were calculated and evaluated according to the specified limits from Claes and Heigele and Shefelbine et al. respectively (13, 26). Figure 5A shows the result of the biomechanical FE simulation for the current treatment. It can be seen very well that about 98 percent of the total 57,466 tetrahedral elements are located in the area that has good mechanical properties for healing and bone formation. Only two percent are in the range between bone resorption and bone formation, while in the remaining ranges there are only single elements, which can also be due to numerical artifacts. The results of the first failed treatment shown in Figure 5B are in clear contrast to this. Here, with 20,773 elements, around 28 percent of the total of 74,977 elements are in the range that was identified as too large. In other words, the first treatment configuration resulted in too much interfragmentary movement latitude in the given fracture morphology. This is a strong indication why the patient did not recover in this setting. The mechanical properties tended to be outside the optimal range to an extent that was too considerable.

## Discussion

Estimating a tibial fracture healing potential based on readily available clinical information remains a challenge, especially immediately after the initial treatment. To identify the risk factors associated with delayed healing several clinical studies have been performed that have shown the overall influence of three general parameters: patient condition, fracture/implant type and morphology and soft tissue integrity/damage (1–3, 17, 30, 31). From these fields, a scoring system has recently been developed to support the surgeons in understanding the non-union risk of a tibial fracture case (2). Despite the scores' limited generalization capability without case-mix adaptation when applied to the SPRINT study outcome data (17), it underlines the relevance of factors associated with vascularity (i.e., presence of compartment syndrome, compromised soft tissue envelope requiring surgical coverage). However, mechanically challenging situations were excluded in the calculation of the score, as the authors did not include cases with a defect situation, or without any cortical apposition. The mechanical environment might not be as relevant in cases with severely compromised soft tissues, or reduced patient condition (i.e., high NURD score due to required flap coverage, compartment syndrome, or immunocompromised host), but in cases with mid-level scores and inconclusive radiography, additional mechanical influence might provide a more specific healing estimation (32). Accordingly, in studies calculating tibial non-union risk under inclusion of fracture morphology characteristics, the results point toward a higher relevance of these parameters in the development of delayed- and non-unions (1, 30, 31). As the patient in our study would have had a non-union risk between 20 and 40% according to the NURD Score, without the possibility to influence any of the factors leading to this risk, we chose to focus on the potentially addressable mechanical fracture environment.

Based on our clinical assessment that the failed initial nailing procedure was due to a mechanical flaw we chose to perform a two-step exchange nailing procedure with an increased nail diameter for the tibia and an additional plate osteosynthesis for the fibula. Exchange nailing is a well-established procedure in aseptic tibial fracture revision cases. It provides an intramedullary autologous bone graft, as well as improved mechanical conditions (33, 34). Our clinical assumption that the failure was due to an insufficient mechanical construct was confirmed by the *post-hoc* simulation results of the failed implant situation in our patient, where the highest amount of hardware strain was seen in the area, where the implant ultimately failed. As the initial fixation failed in a valgus direction, we also chose to perform a fibular plate osteosynthesis as part of the revision. However, the importance of fibula fixation in rather distal tibial fractures has not been clearly defined and biomechanical as well as clinical studies point toward a limited influence on torsional rigidity and maintenance of the resulting stability of the construct in a clinical context (33, 34). Especially when using adequate distal locking, as was performed in our case with the Synthes ASLS screw system as locking screws, previous biomechanical analysis have shown no increasing effect on stability by adding a fibula plate (35). Accordingly, in our analysis, the torsional and shear stress measured was well within the healing thresholds, so we chose to provide no further simulation input concerning the fibula.

The simulations performed focused, as described above, on the mechanical stimulus driven by the strains that occur. It was found that, in the first failed case, both a significantly higher maximum von Mises stress in the implant occurred and significant deviations in the different strains from the limits identified to allow a good bone healing appeared. Together with the high degree of individualization of the chosen approach from the clinical image data including a calibration phantom to motion capturing in a clinical gait setting and the AnyBody-based boundary conditions of the FE simulation, the study provides a good indication why the first chosen treatment caused difficulties and finally had to be revised. The same simulation workflow also provides a clear fingerprint indication of why the current treatment resulted in uneventful clinical healing. The clinical course highlights the advantage of determining mechanical parameters early on as they can potentially be influenced post-operatively by adjusting the weight-bearing behavior of the patient.

### Limitations

This study has several underlying limitations. The determined mechanical conditions cannot be experimentally validated, as there are currently no commercially available intramedullary implants on the market, that could determine the simulated parameters. Apart from the clinical validation through the healed fracture, the general capability of fracture condition simulation has been shown previously (6). Our simulation focuses on the mechanical parameters given by the implant and weight-bearing input only. Biological effects through the reamed exchange nailing, as well as general patient condition (e.g., individual bone mineral density) and vascularity were not part of the simulation, but could potentially influence the healing outcome. In addition, the fibula was not included in the simulation as the calculated shear strains during the simulation with the tibia alone were already well within the clinically acceptable threshold to warrant the increased technical effort.

### Conclusion

In this proof-of-concept study we were able to establish a simulation workflow to determine the mechanical environment of a fracture and fixation situation, predicting clinically confirmed implant mechanical stresses, interfragmentary movement, as well as the resulting volumetric strain and octahedral shear strain based on the patient-specific weight-bearing input. While it is associated with an increased technical effort, it is possible to estimate relevant biomechanical boundary conditions from readily available clinical data. This is possible at a time point when current standard routine diagnostics cannot reliably predict the clinical and functional outcome. Our results warrant further evaluation of simulation-assisted fracture monitoring as part of clinical routine treatment, as it could determine mechanically critical situations at an early time point after surgery and allow for an immediate adaptation of treatment.

## Data Availability Statement

The original contributions presented in the study are included in the article, further inquiries can be directed to the corresponding author.

## Ethics Statement

The studies involving human participants were reviewed and approved by Ethikkommission der Ärztekammer des Saarlandes. The patients/participants provided their written informed consent to participate in this study. Written informed consent was obtained from the individual(s) for the publication of any potentially identifiable images or data included in this article.

## Author Contributions

TP, BB, MO, and MR conceptualized the study, supervised its conduct (investigation), and prepared the manuscript. TP, BB, MO, and SD secured funding. BB, MO, and TP were involved in the clinical treatment and decision process. AB and JG provided and prepared the clinical imaging for further simulation (resources and investigation). KW, AA, MR, and SD performed the movement analysis and simulation (formal analysis). All authors contributed to the manuscript and approved the final version.

## Funding

This study was funded by the Werner Siemens Stiftung.

## Conflict of Interest

The authors declare that the research was conducted in the absence of any commercial or financial relationships that could be construed as a potential conflict of interest.

## Publisher's Note

All claims expressed in this article are solely those of the authors and do not necessarily represent those of their affiliated organizations, or those of the publisher, the editors and the reviewers. Any product that may be evaluated in this article, or claim that may be made by its manufacturer, is not guaranteed or endorsed by the publisher.
